# Supplementary material for: Depressive-like Behaviors Induced by mGluR5 Reduction in 6xTg in Mouse Model of Alzheimer’s Disease
Source: Int J Mol Sci. 2023 Aug 21;24(16):13010. doi: 10.3390/ijms241613010 (PMC10455602; doi:10.3390/ijms241613010)
Supplement: Supplementary file 1 [file ijms-24-13010-s001.zip › ijms-2529936-supplementary.pdf]

International Journal of
Molecular Sciences

Supplementary Materials

Depressive-like Behaviors Induced by mGluR5 Reduction in 6xTg in Mouse Model of Alzheimer's Disease

Youngkyo Kim [1], Jinho Kim [1], Shinwoo Kang [2,3] and Keun-A Chang [1,2,4,*]

1 Department of Health Science and Technology, Gachon Advanced Institute for Health Sciences & Technology, Gachon University, Incheon 21999, Republic of Korea
2 Department of Pharmacology, College of Medicine, Gachon University, Incheon 21999, Republic of Korea
3 Department of Molecular Pharmacology and Experimental Therapeutics, Mayo Clinic, Rochester, VT 55905, USA
4 Neuroscience Research Institute, Gachon University, Incheon 21565, Republic of Korea
* Correspondence: keuna705@gachon.ac.kr

Supplementary Figures

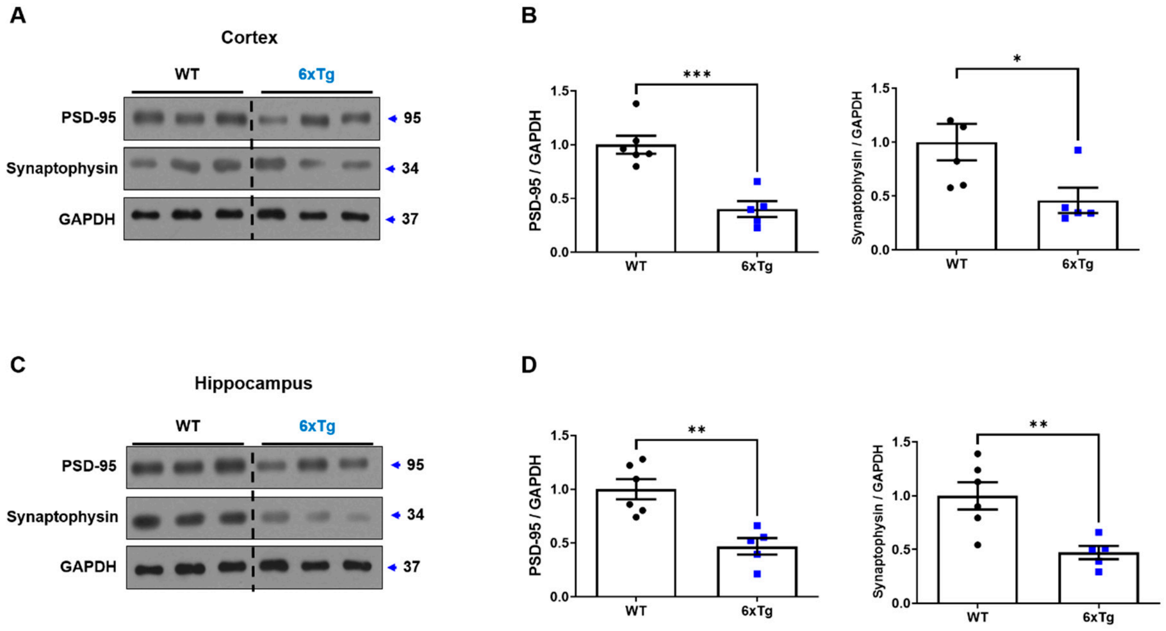

Figure S1. Increased synaptic loss in 8-month-old 6xTg mice

(A) Representative Western blot images depicting PSD-95 and synaptophysin protein levels in the cortex (CX) of 8-month-old WT and 6xTg brains. (B) Quantitative analysis of PSD-95 and synaptophysin expressions in the CX. (C) Representative Western blot images of PSD-95 and synaptophysin protein levels in the hippocampus (HP) of WT and 6xTg brains. (D) Quantitative analysis of PSD-95 and synaptophysin expressions in the HP. Data are presented as means ± SEM (n = 5-6 per group). *$p < 0.05$, **$p < 0.01$, ***$p < 0.001$ vs. WT. Statistical significance between the two groups was determined using the Student t-test.

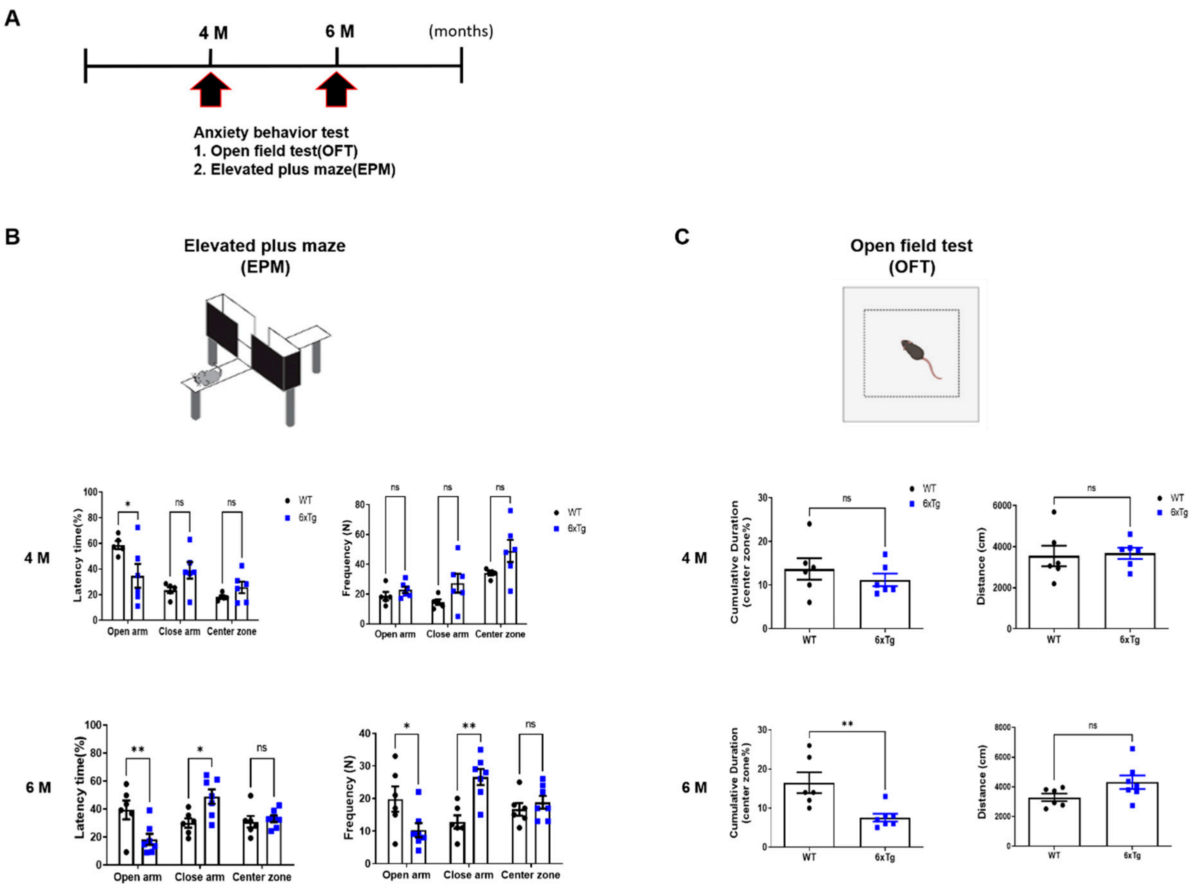

Figure S2. Increased anxiety-like behavior in 6-month-old 6xTg mice.

(A) The Elevated Plus Maze (EPM) and Open Field Test (OFT) were conducted to confirm anxiety-like behaviors according to the experimental schedule. (B) In Elevated Plus Maze (EPM) showed a significant increase in latency times in the closed arm starting from 4 months when comparing WT and 6xTg mice. Data are presented as means ± SEM (n = 6- 7 per group). **p* < 0.05, ***p* < 0.01 vs. WT by two-way ANOVA followed by Bonferroni`s post hoc test. (C) In the Open Field Test (OFT), the 6xTg mice group at 6 months of age displayed significantly increased latency times in the center zone compared to WT mice, indicating higher levels of anxiety-like behavior. Data are presented as means ± SEM (n = 6- 7 per group). ***p* < 0.01 vs. WT. Statistical significance between the two groups was determined using the Student t-test.

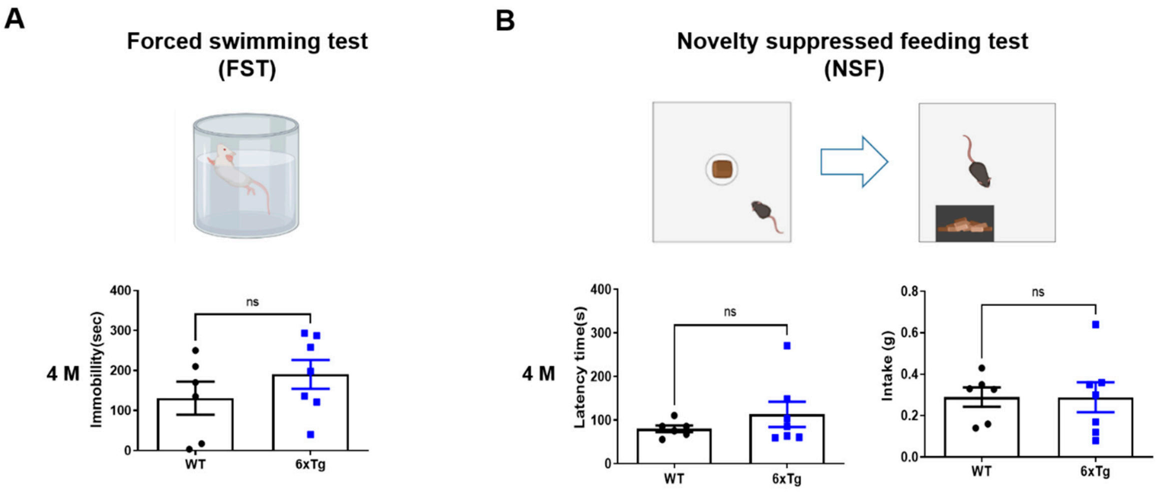

Figure S3 Depressive-like behaviors in 4-month-old 6xTg mice.

(A) The Forced swimming test (FST) and (B) novelty-suppressed feeding test (NSF) were conducted to confirm depressive-like behaviors. Data are presented as means ± SEM (n = 6-7 per group). Statistical significance between the two groups was determined using the Student t-test.

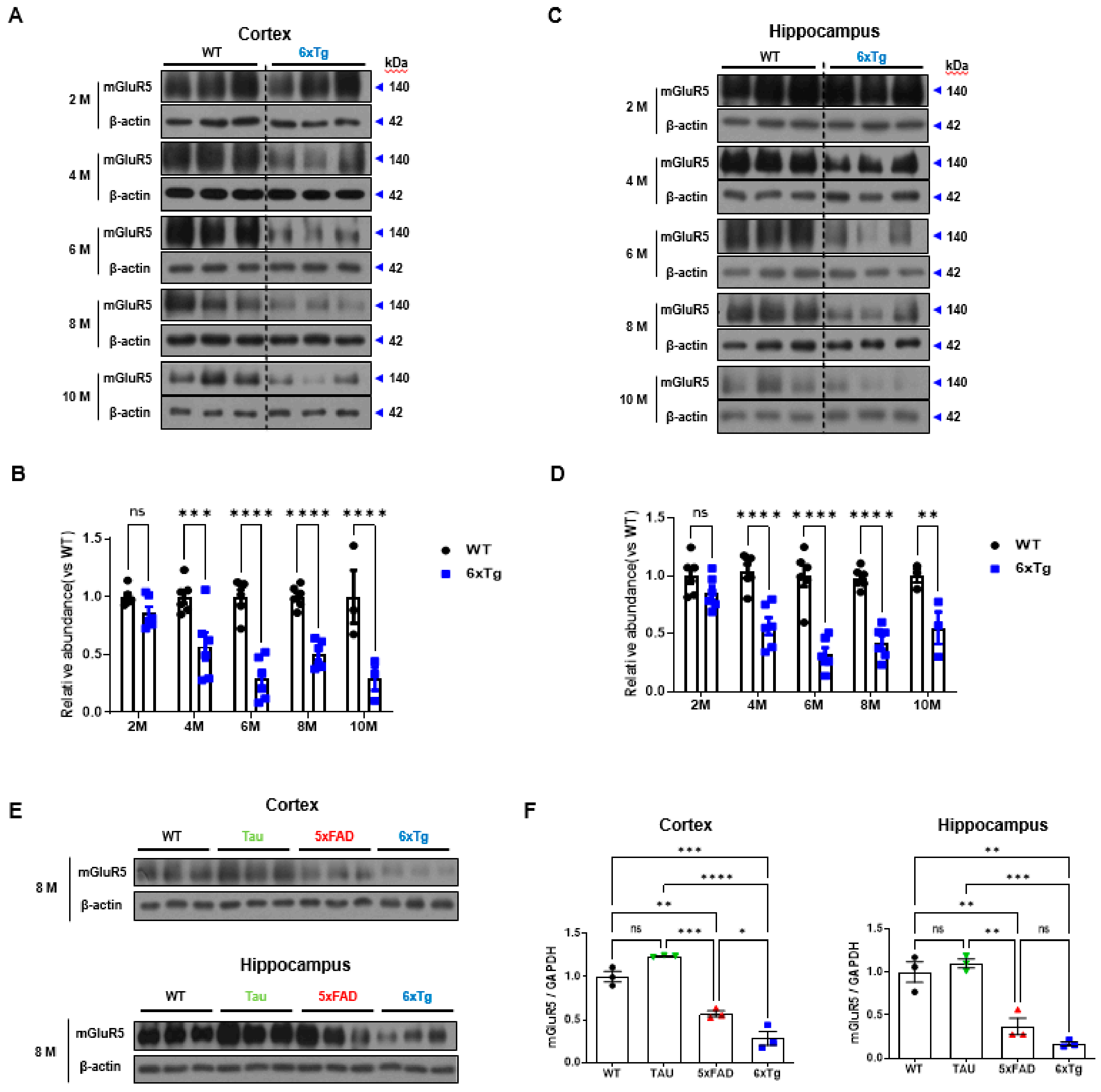

Figure S4 Comparison of the protein levels of mGluR5 between the WT and 6xTg mice.

(A) Representative Western blot image of mGluR5 protein levels in cortex of 2-, 4-, 6- and 8-month-old WT and 6xTg brain. (B) Quantitative analysis of mGluR5 expressions in the cortex. (C) Representative Western blot image of mGluR5 protein levels in hippocampus of 2-, 4-, 6- and 8-month-old WT and 6xTg brain. (D) Quantitative analysis of mGluR5 expressions in the hippocampus. Data are presented as means ± SEM (n = 6 per group). $^{**}p < 0.01$ $^{***}p < 0.001$, $^{****}p < 0.0001$ vs. WT by two-way ANOVA followed by Bonferroni`s post hoc test (E) Representative Western blot images of mGluR5 protein levels in cortex and hippocampus of 8-month-old WT, Tau, 5xFAD and 6xTg mice. (F) Quantitative analysis of mGluR5 expressions in the cortex and hippocampus. Data are presented as means ± SEM (n = 3-6 per group). $^{**}p < 0.01$, $^{***}p < 0.001$ vs. WT, $^{*}p < 0.05$, $^{**}p < 0.01$, $^{***}p < 0.001$, $^{****}p < 0.0001$ vs. Tau, $^{*}p < 0.05$ vs 5xFAD by one-way ANOVA followed by Tukey's multiple comparisons test.

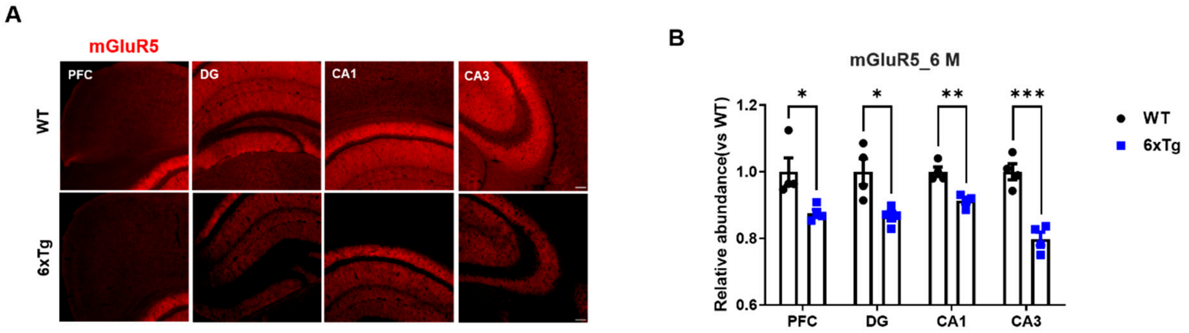

Figure S5 Reduced GluR5 density in brains of 6-month-old 6xTg mice.

(A) Representative IHC images of mGluR5 protein levels in the PFC, DG, CA1, and CA3 of 6-month-old WT and 6xTg brains. Scale bar 100μm (B) Quantitative analysis of mGluR5 expressions in the PFC, DG, CA1, and CA3. Data are presented as means ± SEM (n = 3 per group). *$p < 0.05$, **$p < 0.01$, ***$p < 0.0001$ vs. WT. Statistical significance between the two groups was determined using the Student t-test.

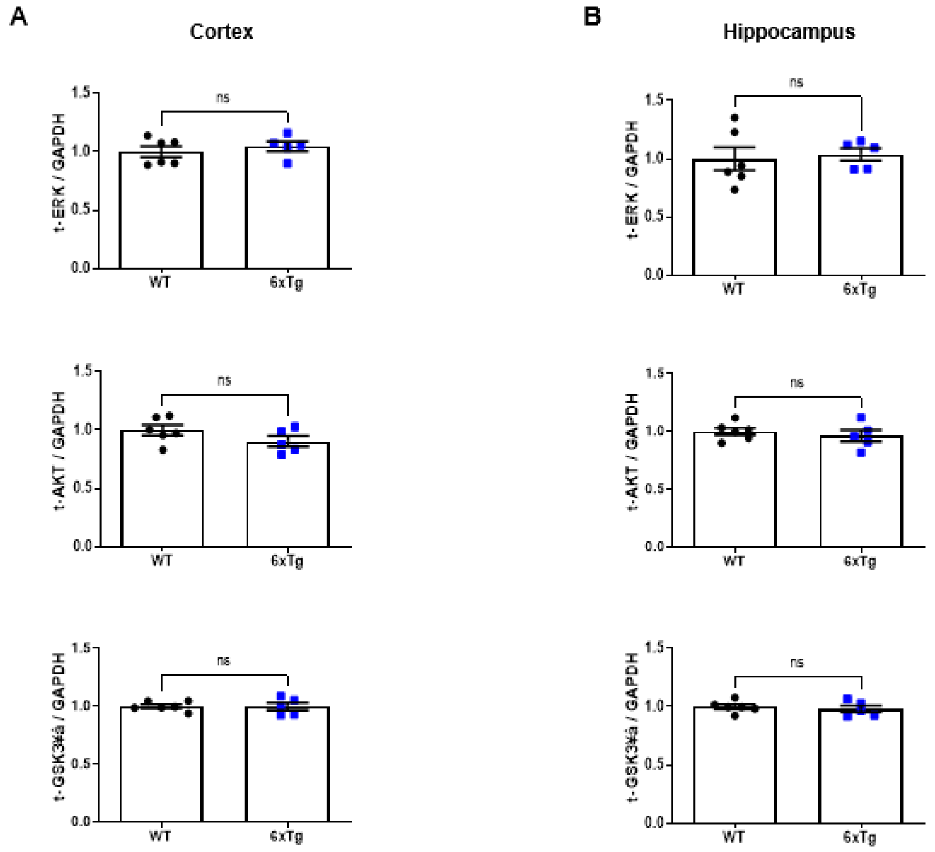

Figure S6. Protein levels of t-ERK, t-AKT, and t-GSK3β in brains of 8-month-old 6xTg mice.

(A) Quantitative analysis of t-ERK, t-AKT, and t-GSK3β expressions n the cortex (CX) of 8-month-old WT and 6xTg mice. (B) Quantitative analysis of t-ERK, t-AKT, and t-GSK3β expressions in the hippocampus (HP) of 8-month-old WT and 6xTg mice. Data are presented as means ± SEM (WT, n = 6; 6xTg, n =6). Statistical significance between the two groups was determined using the Student t-test.
